# Bare carbon electrodes as simple and efficient sensors for the quantification of caffeine in commercial beverages

**DOI:** 10.1098/rsos.172146

**Published:** 2018-05-02

**Authors:** Luca Redivo, Miroslav Stredanský, Elisabetta De Angelis, Luciano Navarini, Marina Resmini, Ĺubomír Švorc

**Affiliations:** 1 Department of Chemistry and Biochemistry, School of Biological and Chemical Sciences, Queen Mary University of London, Mile End Road, London E1 4NS, UK; 2 Biorealis s.r.o., Radlinského 9, 811 07 Bratislava, Slovak Republic; 3 illycaffè S.p.A, via Flavia 110, 34147 Trieste, Italy; 4 Institute of Analytical Chemistry, Faculty of Chemical and Food Technology, Slovak University of Technology in Bratislava, Radlinského 9, Bratislava 812 37, Slovak Republic

**Keywords:** glassy carbon electrode, voltammetry, food quality control, caffeine

## Abstract

Food quality control is a mandatory task in the food industry and relies on the availability of simple, cost-effective and stable sensing platforms. In the present work, the applicability of bare glassy carbon electrodes for routine analysis of food samples was evaluated as a valid alternative to chromatographic techniques, using caffeine as test analyte. A number of experimental parameters were optimized and a differential pulse voltammetry was applied for quantification experiments. The detection limit was found to be 2 × 10^−5^ M (3σ criterion) and repeatability was evaluated by the relative standard deviation of 4.5%. The influence of sugars, and compounds structurally related to caffeine on the current response of caffeine was evaluated and found to have no significant influence on the electrode performance. The suitability of bare carbon electrodes for routine analysis was successfully demonstrated by quantifying caffeine content in seven commercially available drinks and the results were validated using a standard ultra-high performance liquid chromatography method. This work demonstrates that bare glassy carbon electrodes are a simple, reliable and cost-effective platform for rapid analysis of targets such as caffeine in commercial products and they represent therefore a competitive alternative to the existing analytical methodologies for routine food analysis.

## 1. Introduction

Novel technological developments applicable to the area of food quality and safety are driven by strong public interests [1] as well as by the growing numbers of new regulations introduced by food agencies, to ensure that standards are upheld in commercialized products [2]. Most analytical techniques and protocols developed for these purposes allow sensitive and precise quantitative analysis, and rely on equipment-based systems such as capillary electrophoresis (CE) [3,4], gas chromatography (GC) [5,6], high-pressure liquid chromatography (HPLC) [7,8], infrared (IR)-Raman spectroscopy [9,10], surface-enhanced Raman spectroscopy (SERS) [11,12], nuclear magnetic resonance (NMR) [13,14] and UV spectroscopy [15,16]. These techniques are accurate and selective, but require expensive instruments and highly trained workers and in some cases additional steps due to sample pre-treatment [17]. As a result there is a strong demand for new systems characterized by minimal sample pre-treatment, short analysis time, long-term stability and low costs, while not requiring hazardous chemicals or specialized technical support, and maintaining high analytical performance [18,19].

Among the most promising approaches, electrochemistry has grown in interest, having been shown to satisfy most requirements [20–23]. In particular, the development of modified electrodes starting from bare carbon material (glassy carbon, boron-doped diamond, graphene, and screen-printed carbon electrodes) has led to important results and interesting applications [24–27]. The surface modification is key in obtaining excellent performance in terms of sensitivity and specificity, although the significant additional costs, both in terms of added labour and final price of the device represent a limiting factor. Moreover, the short-term stability and the reproducibility of the preparation of modified electrodes has been highlighted recently as an important issue, which discourages further applications especially in industry [28–30]. Bare electrodes, without functionalization, represent an interesting alternative, in particular when high sensitivity is not required. This approach makes use of a simpler system, resulting in reduced costs for both production and use, and a demonstrated long-term stability [31]. Among the different types of bare electrodes, glassy carbon electrodes (GCE) are the ones that have been extensively studied thanks to the relative low cost, chemical inertness and wide anodic potential window [32,33]. Some successful examples of an electrochemical sensor based on bare GCE were recently reported in the literature, in particular in the area of pharmaceutical formulation analysis [34–37].

In this work we explored the potential application of glassy carbon electrodes in quality controls applied to the food industry, and for this purpose caffeine was identified as the model analyte. Caffeine (CAF) is mainly found in coffee, where its concentration varies significantly depending on the type of bean, the degree of roasting, the type of brewing and temperatures used [38,39]. However, in recent years there has been a significant increase in the number of drinks commercially available which contains caffeine, consumed not only by adults but by an increasing number of younger people. CAF is a physiological stimulant acting as adenosine A-receptor antagonist, which has been shown to have both positive and negative effects on health [40]. When the intake of CAF is moderate (less than 400 mg per day), there are positive effects, such as higher concentration and decreased tiredness [41], and in such doses its use has also been documented for the treatment of respiratory diseases [42]. However, in high dosage CAF may cause cardiovascular and calcium balance problems [43], and there is still a debate on what can be considered a safe amount of CAF intake, especially for children and young adults [44]. In other commercially available drinks the content of caffeine can vary significantly, ranging from 0.30 g l^−1^ in energy drinks, or 0.1 g l^−1^ in cola-based drinks or as active ingredient in tablets (usually 200 mg/tablet) or excipient (65 mg/tablet). Given the widespread presence, use and the biological effects of CAF, the routine analysis of its content in all commercialized drinks is a primary task for their manufacturers.

In the present work we demonstrate the validity of using bare GCE for the detection and quantification of CAF content in a variety of commercial samples. The assay is simple, innovative and efficient and most significantly is highly reproducible; the assay was validated with ultra-high performance liquid chromatography.

## 2. Material and methods

### 2.1. Chemicals and reagents

Caffeine, theophylline, theobromine, paraxanthine, glucose, sucrose, formic acid and acetonitrile were purchased from Sigma-Aldrich with analytical grade purity. Double distilled water with resistivity above 18 MΩ cm was employed in all experiments. Sulfuric acid (ACS reagent, 95.0–98.0%), nitric acid (ACS reagent, ≥69%), perchloric acid (ACS reagent, 70%) were tested as supporting electrolytes for CAF sensing. CAF containing beverages were purchased from the local store. Coffee samples were prepared by using medium roasting degree coffee (100% Coffea arabica L. blend) Iperespresso capsule (illycaffè S.p.A., Trieste, Italy). Iperespresso coffee machine (mod. X2, illycaffè S.p.A., Italy) and tap water (total hardness 18–20°f) were used to prepare three different types of espresso beverages according to the typical Italian cup volume known as ristretto, regular or lungo [45].

### 2.2. Apparatus

Electrochemical measurements were conducted in a three-electrode single compartment glass cell using Ag/AgCl (3 M KCl) as reference electrode, platinum as counter electrode and bare glassy carbon electrode (GCE, MetrohmAutolab B.V., The Netherlands) with 2** **mm active surface area as working electrode. The experiments were performed using a small electrochemical analyser 910 PSTATmini (MetrohmAutolab B.V., The Netherlands). The data were collected, handled and analysed using the PSTAT 1.0 (MetrohmAutolab B.V., The Netherlands) and OriginPro 7.5 (OriginLab Corporation, USA) softwares, respectively. The USC100TH ultrasonic bath (VWR, United Kingdom) was used for degassing.

### 2.3. Measurement procedures

Cyclic voltammetry (CV) and differential pulse voltammetry (DPV) techniques were used for the study of the electrochemical behaviour of CAF and its reliable determination as well as for real sample analysis. Firstly, in order to get the active surface area of the GCE clean, the standard procedure based on using polishing with alumina (average grain size of 0.3** **µm) was performed followed by an electrochemical preconditioning of the electrode at +2.0** **V for 30** **s, prior to launching a measurement.

To select the appropriate supporting electrolyte for CAF sensing, three different strong acids in a concentration range from 0.01 up to 0.5 M were tested, including H_2_SO_4_, HNO_3_ and HClO_4_, with a fixed CAF concentration of 0.546** **mM. Two consecutive cyclic voltammograms were recorded each time and the second measurement was the one used. For the optimization of the DPV parameters, the pulse potential, pulse time and scan rate were investigated from 10 to 200** **mV, from 5 to 150** **ms and from 2 to 30** **mV s^−1^, respectively.

A calibration curve was built by subsequent additions of an appropriate volume of caffeine stock solution (10** **mM, in Milli-Q water) in the electrochemical cell where 20 ml of H_2_SO_4_ 0.1 M were present as supporting electrolyte. The caffeine concentration range evaluated was from 3 to 2725 µM, and for each addition three consecutive DPV experiments were done.

### 2.4. Real samples analysis

All samples were analysed without any previous dilution or filtration steps. For carbonated soft drinks (i.e. Coca-Cola, Pepsi-Cola, Kofola, Red Bull) the samples were degassed for 3 min prior to analysis, using ultrasonic bath. The DPV measurements were performed into an electrochemical cell where 20 ml of supporting electrolyte were present and an appropriate volume of the particular beverage was added, i.e. 4 ml for soft drinks (Coca-Cola, Pesi-Cola, Kofola), 1 ml for Red-Bull, and 0.2 ml in the case of espresso brews. The content of CAF was determined using standard addition method, by three consecutive additions of 0.8 ml of a standard stock solution of caffeine (10** **mM in Milli-Q water). After each addition 5 consecutive DP voltammograms were recorded.

### 2.5. Comparative ultra-high performance liquid chromatography method

Ultra-high performance liquid chromatography (UHPLC) was performed using a 1290 Infinity LC system with DAD detector, equipped with a 4.6** **mm × 150** **mm, 2.7 µm 120 SB-C18 Poroshell column, for the analysis of the chromatogram LC OpenLab was used (Agilent Technologies, Waldbronn, Germany). The experimental conditions were the following: the mobile phases were aqueous formic acid (0.1%) and acetonitrile (flow rate equal to 1.2 ml min^−1^), starting at 90% of aqueous phase, reaching 60% at 10** **min, 50% at 12** **min and then back to initial conditions. CAF concentration was determined by monitoring the absorbance at 273** **nm.

The calibration curve was obtained by analysing different CAF solution in Milli-Q water, investigating a concentration range from 0.12 to 1.03** **mM. A good linearity was found within the entire concentration range studied.

For real sample analysis, all beverages were diluted with Milli-Q water 1 : 10 (for energy and soft drinks), 1 : 50 (espresso). The diluted solutions were filtered on Phenex NY 0.2** **µm filters, prior to analysis. The concentration of CAF was determined using the calibration curve, and each sample was analysed five times. The calibration curve equation and an example of the analysis of a caffeinated beverage are reported in the electronic supplementary material.

### 2.6. Data analysis and statistical evaluation

The experimental results were evaluated using OriginPro 7.5 software, and are reported with 95% confidence level interval. For the calibration curve, each point was obtained by the average peak intensity of three consecutive measurements, and the error bar was evaluated based on the standard deviation. The linearity was evaluated using the least-square regression method. The limit of detection (LOD) and limit of quantification (LOQ) were calculated using the 3σ and 10σ criterion, respectively.

With regards to the real sample analysis, in the case of the electrochemical method, the CAF content was determined by interpolation, following the standard addition methodology. For UHPLC analysis, the data are presented as the mean value of 5 repetitions. The error on the CAF content was evaluated according to the following formula:

$$\text{error}=\frac{t_{n-1,\alpha }\times \text{Standard Deviation}}{\text{SQRT}(n)}$$

with *n* = 5, *α* = 0.05, and *t*_4,0.05_ = 2.13.

For the paired *t*-test the QuickCalcs software (GraphPad Software Inc.) was employed. A more exhaustive description of the standard addition methodology and the statistical evaluation is presented elsewhere [46].

## 3. Results and discussion

### 3.1. Electrochemical behaviour study

**Figure 1.**
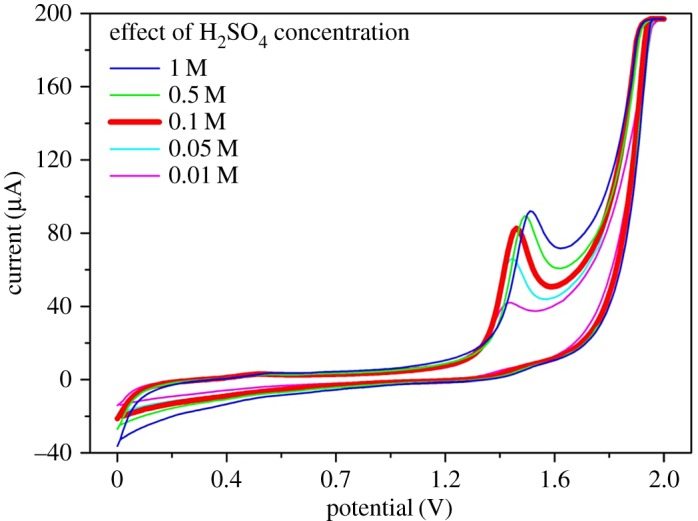
The effect of different concentrations of sulfuric acid for electrochemical oxidation of 0.546 mM CAF on GCE using cyclic voltammetry (scan rate of 100 mV s^−1^).

The first step in the development of an electrochemical sensor is to study the behaviour of the analyte on the electrode's material, and find the best experimental condition (solvent, ionic strength, pH). In particular, the electrochemical behaviour of CAF was previously reported, on bare and modified electrode surfaces, elucidating that the process is highly irreversible, as evidenced by the absence of a reduction signal in the reverse scan [24,31,47]. The process is well known to involve the transfer of four electrons and four protons, leading to the formation of a trimethyl uric acid derivative [48,49]. Generally, an acid medium is employed for electrochemical sensing of CAF. Therefore, in the present work, three strong acids (H_2_SO_4_, HNO_3_, HClO_4_) at different concentrations were evaluated as supporting electrolytes. Figure 1 shows the effect of changes in concentration of H_2_SO_4_, used as supporting electrolyte, in the electrochemical oxidation of CAF using CV. The impact of the different concentrations of HNO_3_ and HClO_4_ is reported in the electronic supplementary material (figures S1 and S2). When different acids were evaluated all at the same concentration of 0.1 M, perchloric and nitric acids performed similarly while in the case of sulfuric acid a narrower and sharper peak was obtained together with a higher S/N ratio (electronic supplementary material, figure S3). A possible explanation for the difference may be found in the variation in ionic strength between the monoprotic acids and the diprotic sulfuric acid. A similar observation has been reported recently by Chalupczok *et al*. [50], using RuO_2_, who conclude that changes in ionic strength have significant impact even when concentrations and pHs of the solutions are the same. As evidenced in figure 1, the favourable voltammetric peak of CAF with a maximum at +1.45** **V was achieved using H_2_SO_4_ at a concentration of 0.1 M. Increases in H_2_SO_4_ concentration resulted in a higher peak for CAF; however, increase in the background current was also noticed. Sulfuric acid was used at 0.1 M as the optimal concentration, as it gave the highest S/N ratio (data shown in electronic supplementary material, table S1 and figure S4). In addition, at high acid concentration (i.e. 1 and 0.5 M) a broad band was observed in the direct scan at 1.6** **V (electronic supplementary material, figure S4). This signal is probably due to the oxidation of carbon atoms from the GCE surface in highly acidic conditions. Previous works in the literature [51–53] have reported that a change in morphology of glassy carbon can be observed when sulfuric acid is employed and long electrochemical pre-treatments applied. However, in this proposed work, no long pre-treatments were done and no electrochemical signals of the oxidation of glassy carbon were recorded, which suggests that oxidation of the glassy carbon electrode is negligible under the experimental conditions used for this work. Additionally, short electrochemical pre-treatment steps were done to improve the repeatability of the oxidation signal. Both positive and negative potentials (−2.00, +1.00, +1.75, +2.00** **V) were tested for different lapses time (results not shown), and the best option was found by applying +2.0** **V for 30** **s.

**Figure 2.**
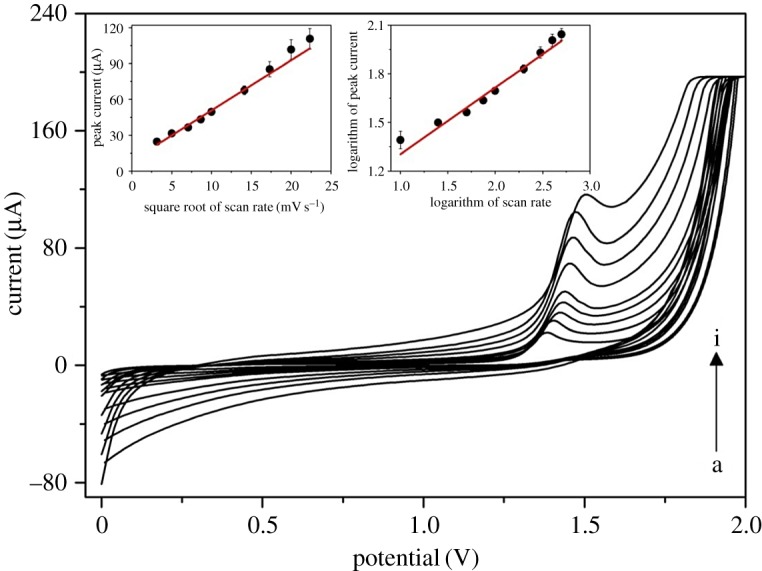
CV records of 1.25 mM CAF for different scan rates (v): (a) 10, (b) 25, (c) 50, (d) 75, (e) 100, (f) 200, (g) 300, (h) 400 and (i) 500 mV s^−1^ in 0.1 M H_2_SO_4_ on GCE. The peak current as a function of square root of the scan rate and logarithmic analysis are appended in the inset.

GCE is well known for adsorbing molecules on its active surface during the electrochemical measurement, usually leading to a decline of the peak of the particular analyte due to the surface passivation by analyte and/or products of its electrode reaction. In our case, in order to assess whether the redox reaction of CAF is controlled by adsorption or diffusion on the GCE, the effect of different scan rates was studied. The scan rate value was examined from 10 up to 500** **mV s^−1^ in 0.1 M H_2_SO_4_ containing 1.25** **mM CAF. Figure 2 reveals that by increasing the scan rate, the oxidation peak shifted towards higher potentials, which is a typical behaviour for electrochemically irreversible systems. The linear relationship between the peak current of CAF (in µA) and the square root of the scan rate (mV s^−1^) was noticed (inset of figure 2) with the following regression equation (equation (3.1)):

3.1 $$\text{Peak current }(\mu \text{A})=5.8(\pm 1.8)+4.2(\pm 0.2)\times \text{square root of scan rate}\;R^{2}=0.984.$$

The low intercept value and good linearity indicate that the redox process of CAF on GCE is predominantly driven by diffusion. In addition, a plot of the logarithm analysis (inset of figure 2, equation (3.2)) appeared to be linear, with the slope value of 0.41, in close conformity with the theoretical value (0.50) for a diffusion-controlled process [54].

3.2 $$\text{Peak current}\;(\mu \text{A})=0.89(\pm 0.06)+0.41(\pm 0.03)\times \log_{10}\;\text{scan rate}\;R^{2}=0.975.$$

Thus, the effect of adsorption in electrochemical CAF sensing may be considered as minor, even when using GCE as the working electrode.

### 3.2. Analytical performance evaluation

**Figure 3.**
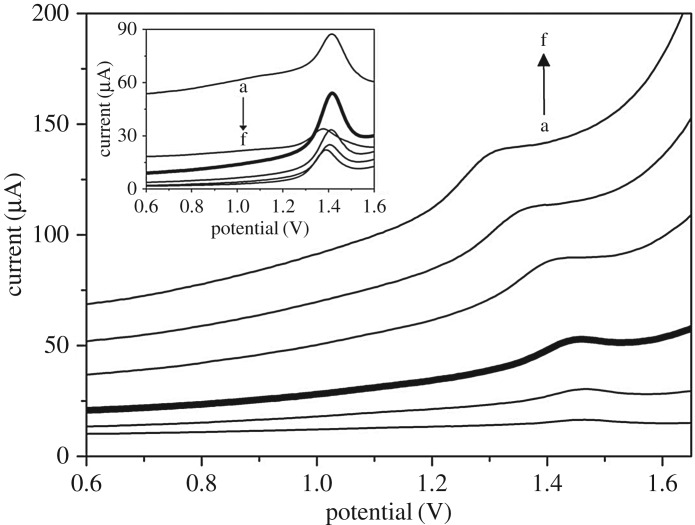
DPV records of 0.44 mM CAF in 0.1 M H_2_SO_4_ on GCE for various pulse potentials: (a) 10, (b) 25, (c) 50, (d) 100, (e) 150 and (f) 200 mV. The optimization of pulse time: (a) 5, (b) 10, (c) 25, (d) 50, (e) 100 and (f) 150 ms appears in the inset.

Prior to constructing the calibration curve for the determination of CAF, the instrumental settings of DPV have to be optimized to achieve the favourable analytical performance. This optimization involves the searching for suitable values of pulse potential (from 10 to 200** **mV), pulse time (from 5 to 150** **ms) and scan rate (from 2 to 30** **mV s^−1^). The results showed that the best compromise between sharpness of the oxidation peak of CAF and measurement period was found with a scan rate of 30** **mV s^−1^. With regards to the pulse potential (figure 3), its increase gave rise to the growth and widening of the oxidation signal of CAF and at the same time the background current sharply increased. Hence, the best setting was found to be 50** **mV. The effect of the pulse time was also studied (inset of figure 3) and 10** **ms was found to be the best value, since further increases caused a decrease in the CAF signal.

Having optimized the instrumental parameters of DPV, the calibration curve for CAF was obtained. It was found that the DPV gave a significant linear relationship of peak current against CAF concentration from 28 to 479 µM (the origin studied range was from 3 up to 2725 µM) according to the following equation (equation (3.3)):

3.3 $$\text{Peak current}\;(\mu \text{A})=1.583(\pm 0.499)+0.091(\pm 0.008)\times \text{caffeine concentration }(\mu \text{M})\;R^{2}=0.992.$$

**Figure 4.**
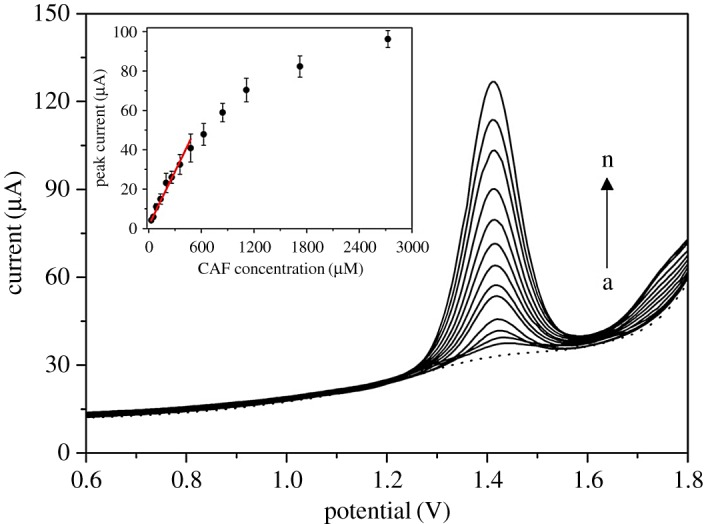
DPV records for various CAF concentrations: (a) 0, (b) 28, (c) 53, (d) 86, (e) 135, (f) 199, (g) 263, (h) 356, (i) 479, (j) 627, (k) 841, (l) 1112, (m) 1724 and (n) 2725 µM in 0.1 M H_2_SO_4_ on GCE. DPV parameters: pulse potential of 50 mV, pulse time of 10 ms and scan rate of 30 mV s^−1^. The dependence between the peak current and CAF concentration is appended in the inset.

The LOD and LOQ were found to be 2 × 10^−5^ and 5 × 10^−5^ M, respectively. The DPV records, demonstrating the distinct oxidation peaks for the various concentrations of CAF with the particular calibration curve, are reported in figure 4. The reproducibility of the method was evaluated by measuring five consecutive CVs of a 1.25** **mM CAF solution with the reached relative standard deviation of peak current of 4.5%. The low RSD value reveals the fact that GCE used in this work provides the precise measurements for CAF sensing. Overall, the analytical performance obtained is suitable for the analysis of commercialized caffeinated products, where CAF content is in the mM range. In addition, the long-term stability of the working electrode is ensured by the high chemical inertness of glassy carbon [32,33].

Numerous analytical techniques and protocols have been previously developed and reported for the detection and quantification of CAF. An overview of the most recent analytical methods for the determination of CAF is presented in table 1, together with the LOD reported for each case.

**Table 1.** Comparison of different analytical techniques for caffeine determination, based on recently reported work.

analytical method	limit of detection	application	references
liquid chromatography	usually in the range of 10^−7^ M but can be as low as 10^−9^ M	different matrix from beverages to biological samples (blood, urine, saliva)	[55–57]
capillary electrophoresis	5 × 10^−5^ M	serum samples	[3]
nuclear magnetic resonance	7 × 10^−7^ M	salivary samples	[58]
surface-enhanced Raman scattering	2 × 10^−6^ M	tertiary solid mixture of paraxanthine theobromine and caffeine	[11]
voltammetry based on bare glassy carbon electrode	2 × 10^−5^ M	caffeinated beverages	this study

Liquid chromatographic methods are the most employed techniques for CAF detection, thanks to the excellent analytical performance (i.e. low sensitivity, wide linear range, selectivity and robustness) and the possibility to be applied with a variety of matrices. Other analytical techniques were investigated for the detection of CAF, like CE, NMR, SERS. All these methods were found suitable for the determination of CAF content and the sensitivity was found to be lower compared to the voltammetric sensor developed in this work. On the other hand, when the focus is on the application of a sensing platform for an industrial application the proposed glassy carbon-based sensor has many advantages, compared with the other methodologies. In fact, our device is small, portable and non-expensive; no hazard or highly pure solvents are required; and no pre-treatments are necessary.

With regards to previously developed electrochemical sensors for CAF they are mostly based on modified GCE and a comprehensive review on this topic was written by Švorc [21]. The functionalization of the surface of GCE was investigated using different types of materials like Nafion [47], metallic nanoparticles [25,59] and polymers [60]. Compared with our bare electrodes, the surface modification enhances the performance in terms of LOD (which can as low as 1 × 10^−9^ M [61]). This high sensitivity is necessary for the analysis of non-caffeinated beverages (where traces of caffeine are still present) or biological samples where the concentration of CAF is low (micromolar range). On the other hand, the long-life stability of the sensing device is an issue, and in many cases the analytical performance is not maintained after one month [26,62,63]. In addition, the chemical modification of the surface has a significant impact on its production and costs, resulting in an increased final price of the sensor, which can limit its widespread applicability.

Therefore in the case of industrial applications involving the routine analysis of commercialized caffeinated beverages, modified electrodes although sensitive and precise are not suitable choices in terms of long-life stability and costs. On the other hand, the proposed bare GCE is a suitable alternative to the currently employed techniques, thanks to its adequate sensitivity at the required concentrations, higher simplicity and high long-term stability. Furthermore, the electrode-based sensor has been used regularly over a period of several months for the quantification of caffeine content in coffee samples, and no significant variations in its performance were observed, suggesting that in the conditions employed any changes in the morphology and properties of the glassy carbon electrode are not significant.

### 3.3. Interference study

The next step in the work focused on the evaluation of the specificity of the electrode when operating in the presence of interfering agents frequently found in commercialized drinks. In fact, common caffeinated beverages, such as coffee and soft drinks, contain other chemical species, which may interfere with the signal of CAF, thus significantly affecting the reliability of method. The study focused on the effect of glucose and sucrose, as these compounds are commonly present in various beverages. Although these substances are not usually oxidized on bare carbon electrodes, they may be adsorbed onto the working electrode surface, thus considerably affecting the analyte signal. The results revealed that the peak potential and shape of CAF signal are not substantially influenced by the presence of these substances up to 100 times higher concentration (electronic supplementary material, figures S5 and S6). However, a moderate change in the background current was recorded, especially in the case of glucose, which is probably due to its adsorption on the surface of the working electrode.

Subsequently, selectivity studies to evaluate the specificity of the detection towards structurally related compounds, such as theophylline, theobromine and paraxanthine, were also performed. Electronic supplementary material, figures S7 and S8 display the effect of the presence of theophylline and paraxanthine (both from 1 : 1 up to 1 : 10 concentration ratio), respectively. It was found that the oxidation of these dimethyl xanthines occurred at a lower potential compared with CAF (+1.26 and +1.22** **V for theophylline and paraxanthine, respectively). This peak-to-peak separation (towards CAF) led to no differences in the shape and position of CAF signal. On the other hand, the peak current of CAF decreased by approximately 10% when an equimolar concentration ratio between CAF and theophylline was present. In the case of paraxanthine, the oxidation peak of CAF increased negligibly (2%). The selectivity observed thus far is promising and provides important preliminary data for the potential use of GCE for the simultaneous detection and quantification of CAF and paraxanthine and/or theophylline. Moreover, in the case of theobromine, an increase in the CAF signal was observed in an equimolar ratio (electronic supplementary material, figure S9). The DPV scan of a solution of 333.3** **µM theobromine (not shown here) shows that under the experimental conditions, theobromine rendered two oxidation peaks at +1.10 and +1.42** **V, the second one overlapping with CAF signal. Overall, when the concentration ratio between the interfering xanthines and CAF was higher (up to 10 : 1), a partial overlapping of the signals was observed for theophylline; in the case of paraxanthine a decrease of 50% in the intensity was noticed. However, this limitation was not expected to affect the reliable determination of CAF in caffeinated beverages (coffee, soft drinks) where the concentration of theophylline, theobromine and paraxanthine is negligible [64,65] compared to the target analyte. On the other hand, in the case of coffee samples, polyphenols are usually present in similar amount as CAF (2.14** **g l^−1^ in the final brew) [66]. These compounds are electrochemically active; however, their oxidation peak potential (around 0–600** **mV), is different from CAF. Besides, their oxidation is strongly suppressed in acidic conditions [67]. As a consequence, no significant voltammetric peaks attributing to polyphenols and affecting the CAF signal were observed during the analysis of coffee samples (electronic supplementary material, figure S10).

### 3.4. Method validation

**Figure 5.**
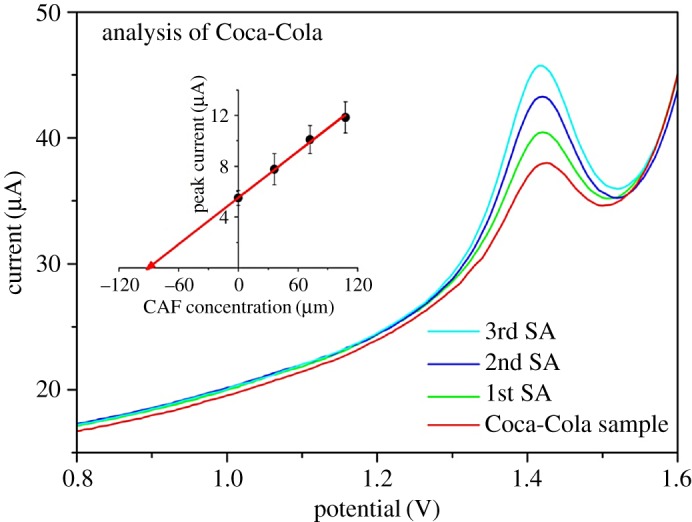
DPV records of analysis of Coca-Cola sample using the standard addition method in 0.1 M H_2_SO_4_ on GCE after addition of 4 ml of sample and after spiking of 80, 160 and 240 µl of 10 mM CAF solution. DPV parameters: pulse potential of 50 mV, pulse time of 10 ms and scan rate of 30 mV s^−1^. The analysis by standard addition method is depicted in the inset.

The validation of the data obtained using bare glassy carbon electrodes was obtained by the analysis of four different commercially available caffeinated beverages and three different espresso brews. The standard addition method was applied for quantification of CAF in order to limit the matrix effect and to prove that the accuracy was within the expected limits. Figure 5 reflects an illustrative example of analysis of Coca-Cola sample using the developed platform. The results were compared with a reference UHPLC method.

The data presented in table 2 clearly indicate that the results obtained by the new method (DPV) are in good agreement with that obtained using the UHPLC method. The employed conditions for the chromatographic analysis are reported in the Material and methods section. The calibration curve was obtained using solutions of caffeine prepared in Milli-Q water and the equation obtained is reported below.

$$\text{Signal area}\;(\text{arb. units})=6.698+4.611\times \text{caffeine concentration}\;(\text{ng}\;\mu {\text{l}}^{-1}).$$

**Table 2.** Real samples analysis of seven different caffeinated beverages (*n* = 5).

	determined CAF content^a^ (g l^−1^)	
commercial caffeinated beverage	proposed DPV	reference UHPLC
regular espresso coffee	3.047 ± 0.142	3.170 ± 0.195
*lungo* espresso coffee	1.709 ± 0.178	1.672 ± 0.153
*ristretto* espresso coffee	4.843 ± 0.291	5.326 ± 0.139
Coca-Cola	0.098 ± 0.010	0.095 ± 0.008
Pepsi-Cola	0.100 ± 0.006	0.110 ± 0.010
Kofola	0.070 ± 0.010	0.063 ± 0.005
Red Bull	0.361 ± 0.097	0.311 ± 0.028

^a^95% confidence interval calculated according [mean ± *t_n_*_−1,_*_α_* s.d./sqrt(*n*)]; *t*_4,0.05_ = 2.13.

A good linearity was found in the concentration range between 0.12 and 1.03** **mM, with an *R*^2^ = 0.9997. In addition, the chromatographic analysis of real samples showed a clear and isolated CAF signal with a retention time of 5.30** **min. An illustrative example of the UHPLC analysis is reported in the electronic supplementary material (figures S11 and S12). Besides, according to the paired *t*-test [46] under 95% confidence level, no statistically significant differences were noticed between the values found by these methods since calculated *t* value (1.04) was lower than the tabulated one (2.45 for *α* = 0.05 and number of measurements *n* = 6). To summarize, no significant interference of the other compounds present in the analysed coffee and soft drink samples was recorded and the developed method provided good accuracy for the determination of CAF in caffeinated beverages. Moreover, the proposed procedure is simple and convenient, since it does not involve any pre-treatment of the samples and can be used as an innovative alternative to conventional analytical methods in CAF assessment and assurance for routine beverage analysis.

## 4. Conclusion

In the present work, bare GCE was evaluated as alternative sensing platform to chromatographic methods for routine analysis in food samples, using CAF as the model analyte. The proposed sensor was shown to allow the precise and selective analysis of CAF content in commercial preparations. The wide applicability of the sensor was assessed by the analysis of seven commercially available caffeinated beverages and the data were found to be in agreement with a standard UHPLC method, routinely employed by a coffee company. The advantages of using the glassy carbon electrode instead of the more complex modified ones are related to the absence of any problems connected with storage and long-term stability. Furthermore, the use of this simple unmodified electrode avoids the use of costly and time-consuming surface functionalization steps. Therefore, bare glassy carbon electrodes, thanks to the chemical inertness, relative low costs and simplicity, can be considered good candidates for the development of accurate and innovative devices for routine quality analysis in the food industry.

## Supplementary Material

Supplementary Material with further experimental results

## Data accessibility

The datasets supporting this article have been uploaded as part of the electronic supplementary material.
