# Supplementary material for: Bare carbon electrodes as simple and efficient sensors for the quantification of caffeine in commercial beverages
Source: R Soc Open Sci. 2018 May 2;5(5):172146. doi: 10.1098/rsos.172146 (PMC5990824; doi:10.1098/rsos.172146)
Supplement: Supplementary Material with further experimental results [file rsos172146supp1.docx]

**SUPPLEMENTARY MATERIAL**

**Simple and efficient electrochemical assay for the determination of caffeine content in beverages using a glassy carbon electrode**

Luca Redivo^a*^, Miroslav Stredanský^b^, Elisabetta De Angelis^c^, Luciano Navarini^c^, Marina Resmini^a^, Ľubomír Švorc^d^

*^a^Department of Chemistry and Biochemistry, School of Biological and Chemical Sciences, Queen Mary University of London, Mile End Road, London, E1 4NS, United Kingdom*

*^b^Biorealis s.r.o., Radlinského 9, 811 07 Bratislava, Slovak Republic*

*^c^illycaffè S.p.A, via Flavia 110, 34147, Trieste, Italy*

*^d^Institute of Analytical Chemistry, Faculty of Chemical and Food Technology, Slovak University of Technology in Bratislava, Radlinského 9, Bratislava, 812 37, Slovak Republic*

__________________________________________________________________________


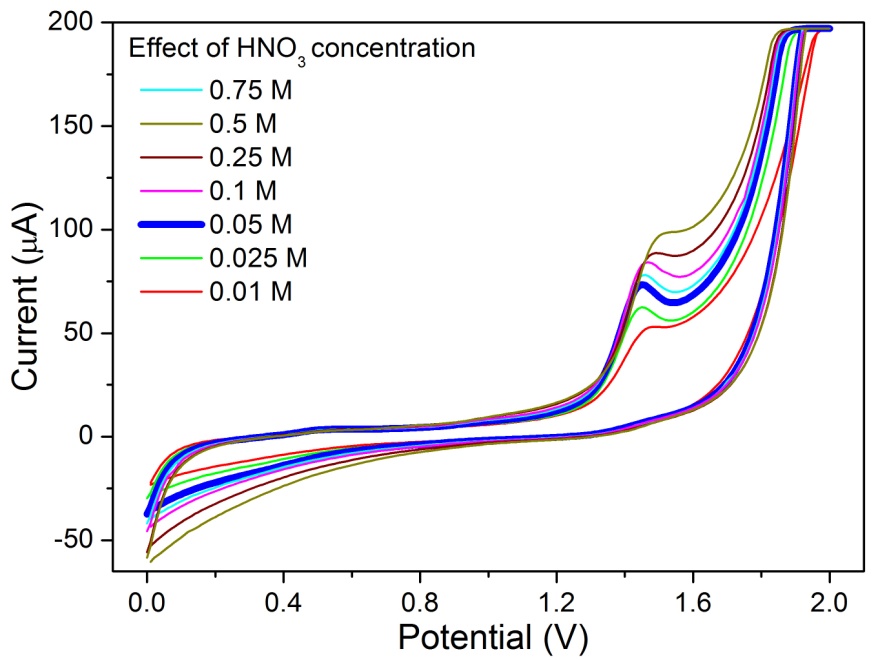


**Fig. S1.** The effect of different concentrations of nitric acid for electrochemical oxidation of 0.546∙mM CAF solution on GCE using CV (scan rate of 100 mV/s)


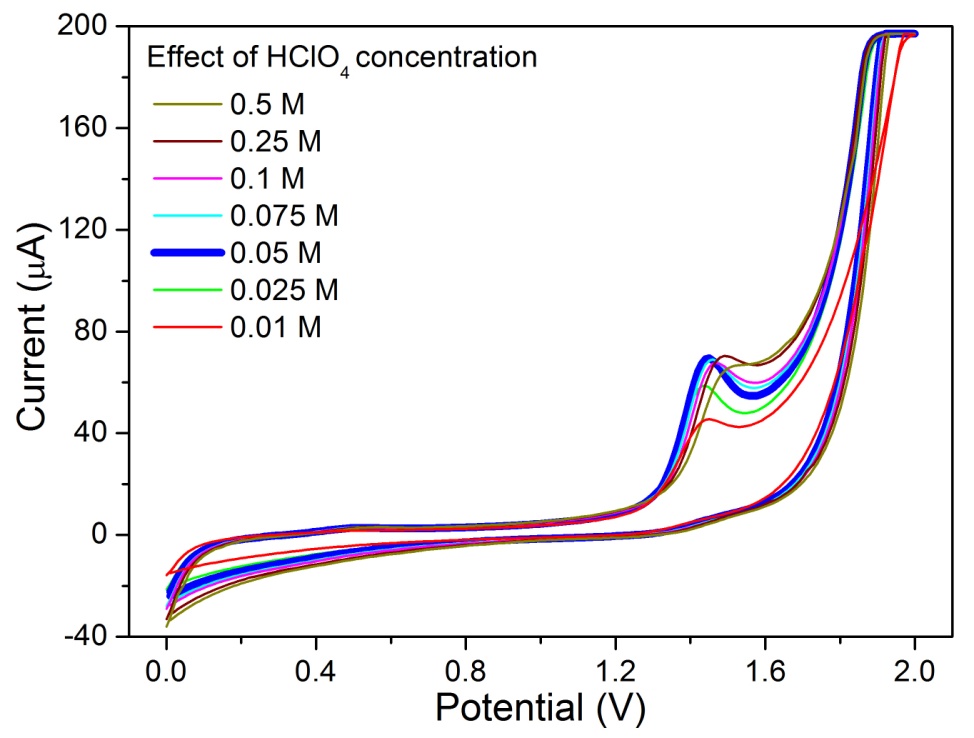


**Fig. S2.** The effect of different concentrations of perchloric acid for electrochemical oxidation of 0.546∙mM CAF solution on GCE using CV (scan rate of 100 mV/s)


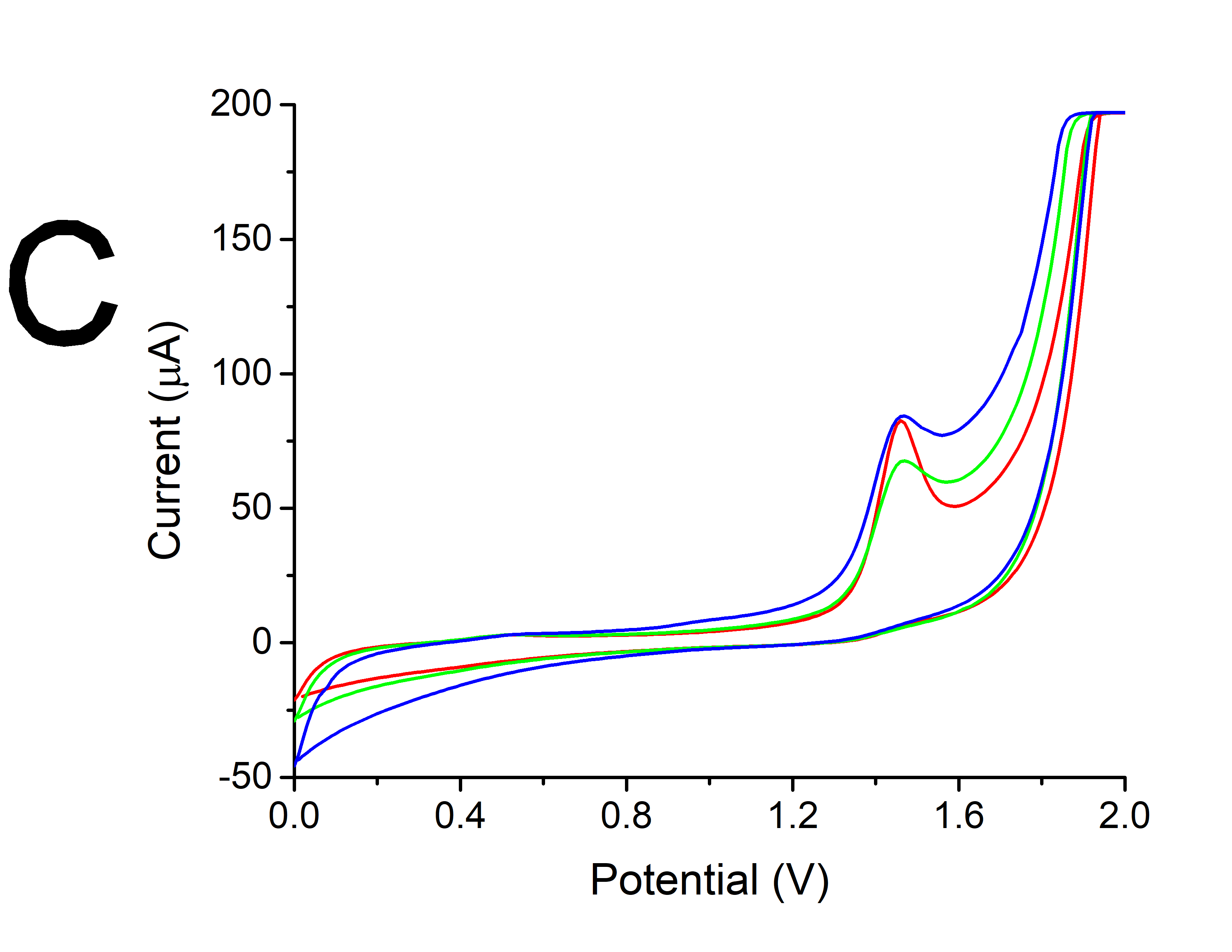


**Fig. S3.** Comparison of the three different acids at the same molar concentration (0.1 M) for electrochemical oxidation of 0.546∙mM CAF solution on GCE using CV (scan rate of 100 mV/s). Red line H_2_SO_4_ (0.1M), green line HClO_4_ (0.1M), Blue line HNO_3_ (0.1M).


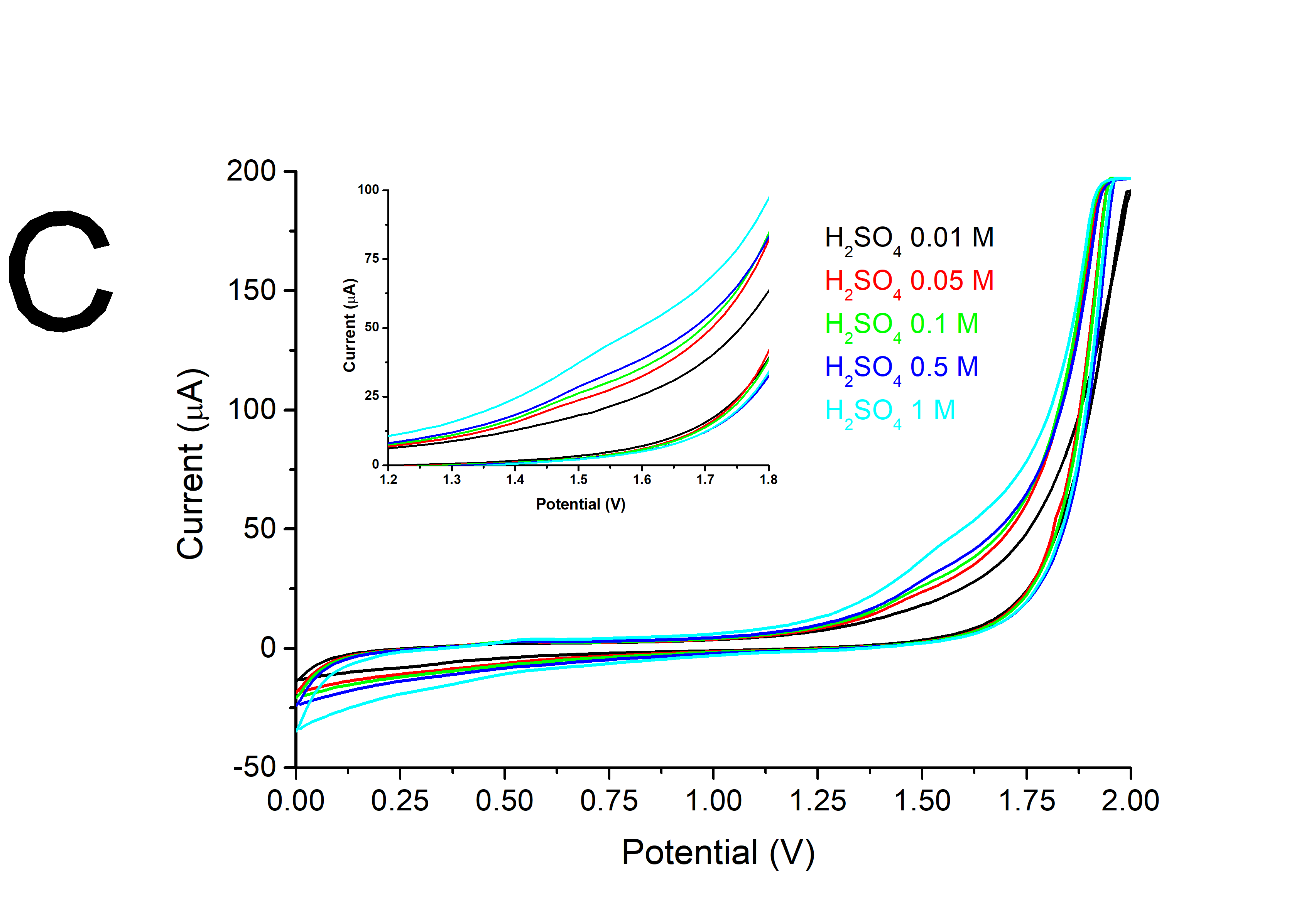


**Fig. S4.** Cyclic voltammograms of different concentrations of H_2_SO_4_ in milli-Q water, all voltammograms were recorded with a scan rate of 100 mV/s. Black line H_2_SO_4_ 0.01 M, red line H_2_SO_4_ 0.05 M, green line H_2_SO_4_ 0.1 M, navy-blue line H_2_SO_4_ 0.5 M, light blue line H_2_SO_4_ 1 M..

**Table. S1.** S/N ratio at different sulphuric acid concentrations. The intensity at the maximum of caffeine oxidation peak was considered for evaluation of the ratio. All experiments were performed using CV with a scan rate of 100 mV/s for a caffeine concentration of 0.546∙mM.

| *H_2_SO_4_ concentration (M)* | S/N *ratio* |
| --- | --- |
| *0.01* | *2.84* |
| *0.05* | *3.34* |
| *0.1* | *3.67* |
| *0.5* | *3.26* |
| *1* | *2.38* |


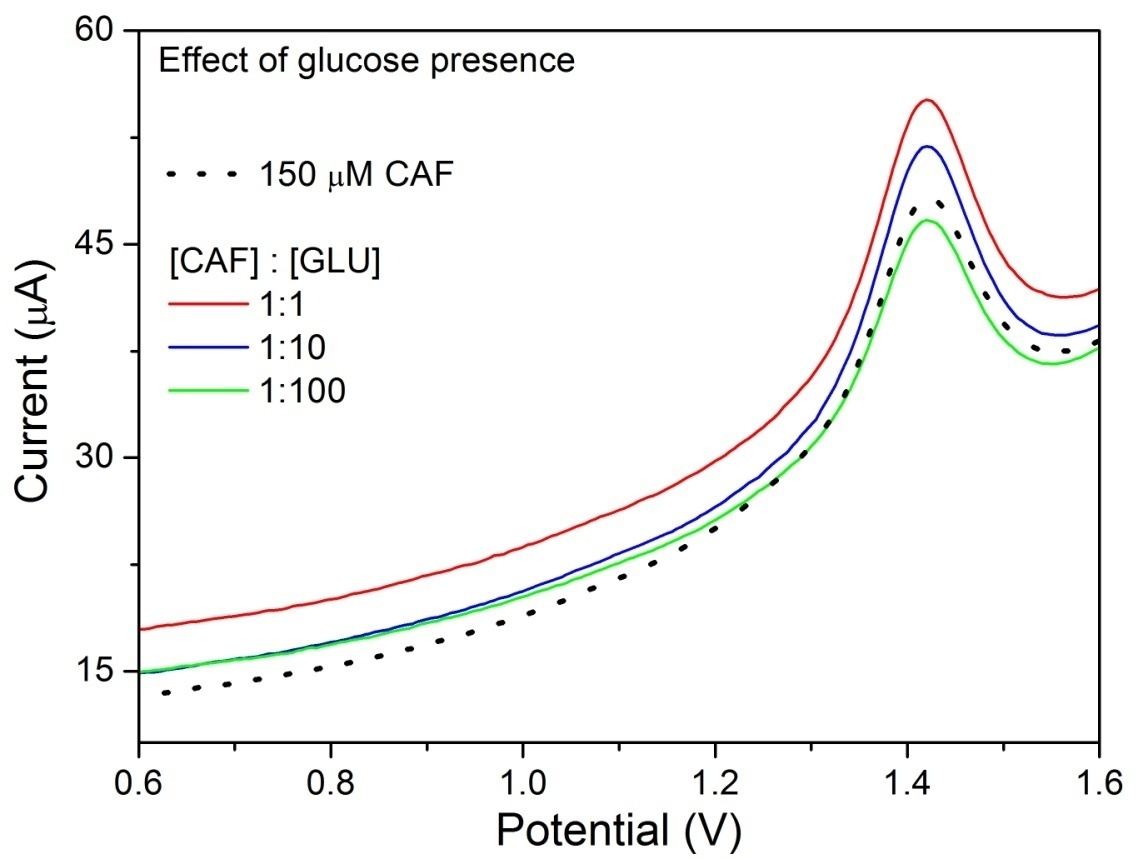


**Fig. S5.** DP voltammograms demonstrating the effect of the presence of glucose (GLU) on current response of 150 µM CAF in 0.1 M H_2_SO_4_ on GCE. The concentration ratios between CAF and GLU are stated in the legend of figure. DPV parameters: pulse potential of 50 mV, pulse time of 10 ms and scan rate of 30 mV/s.


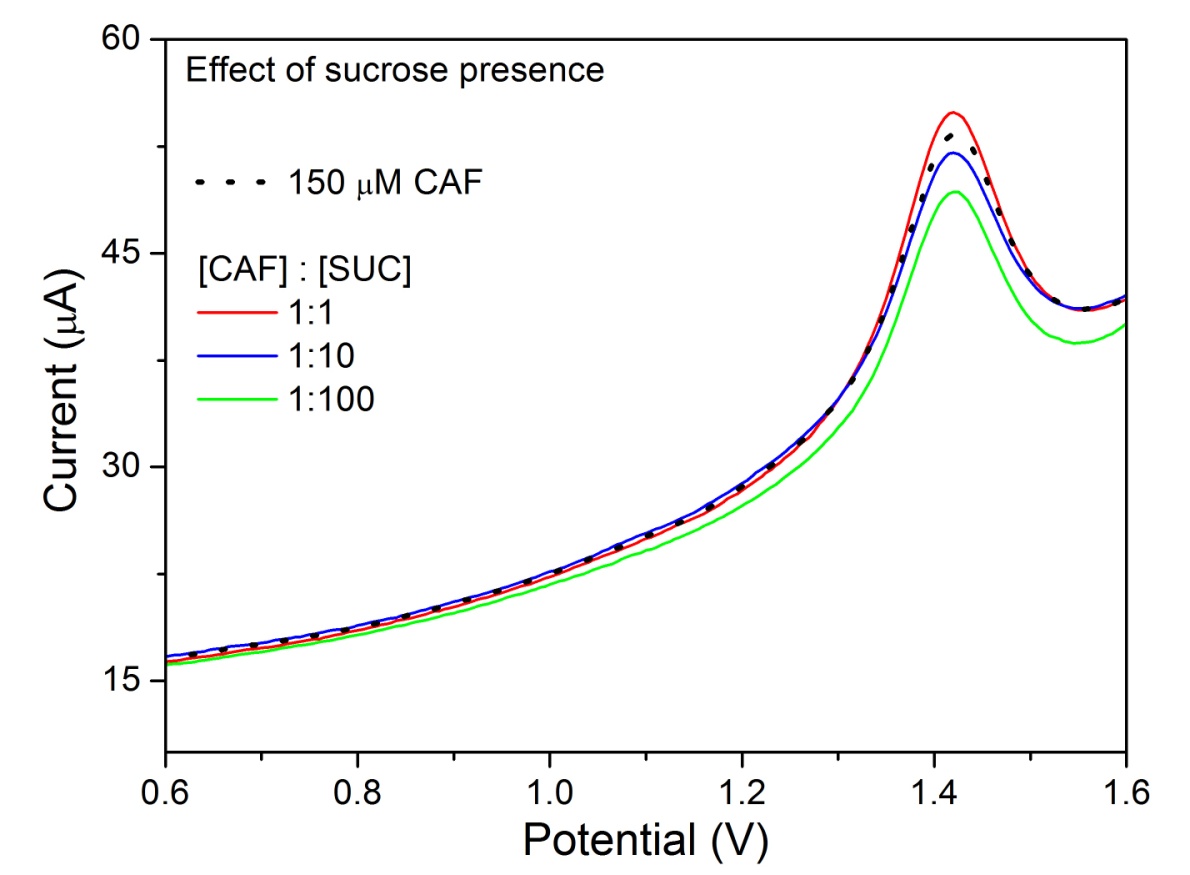


**Fig. S6.** DP voltammograms demonstrating the effect of the presence of sucrose (SUC) on current response of 150 µM CAF in 0.1 M H_2_SO_4_ on GCE. The concentration ratios between CAF and SUC are stated in the legend of figure. DPV parameters: pulse potential of 50 mV, pulse time of 10 ms and scan rate of 30 mV/s.

**
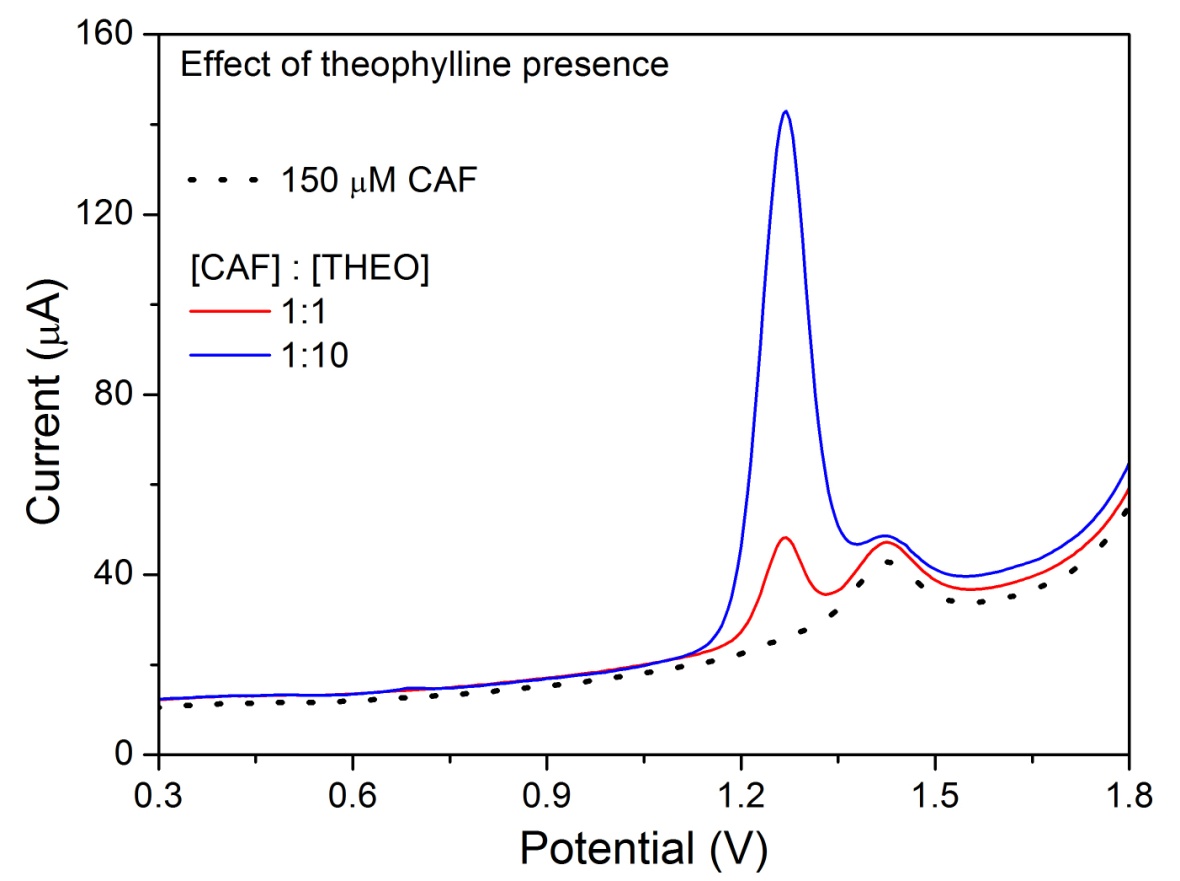
**

**Fig. S7.** DP voltammograms demonstrating the effect of the presence of theophylline (THEO) on current response of 150 µM CAF in 0.1 M H_2_SO_4_ on GCE. The concentration ratios between CAF and THEO are stated in the legend of figure. DPV parameters: pulse potential of 50 mV, pulse time of 10 ms and scan rate of 30 mV/s.

**
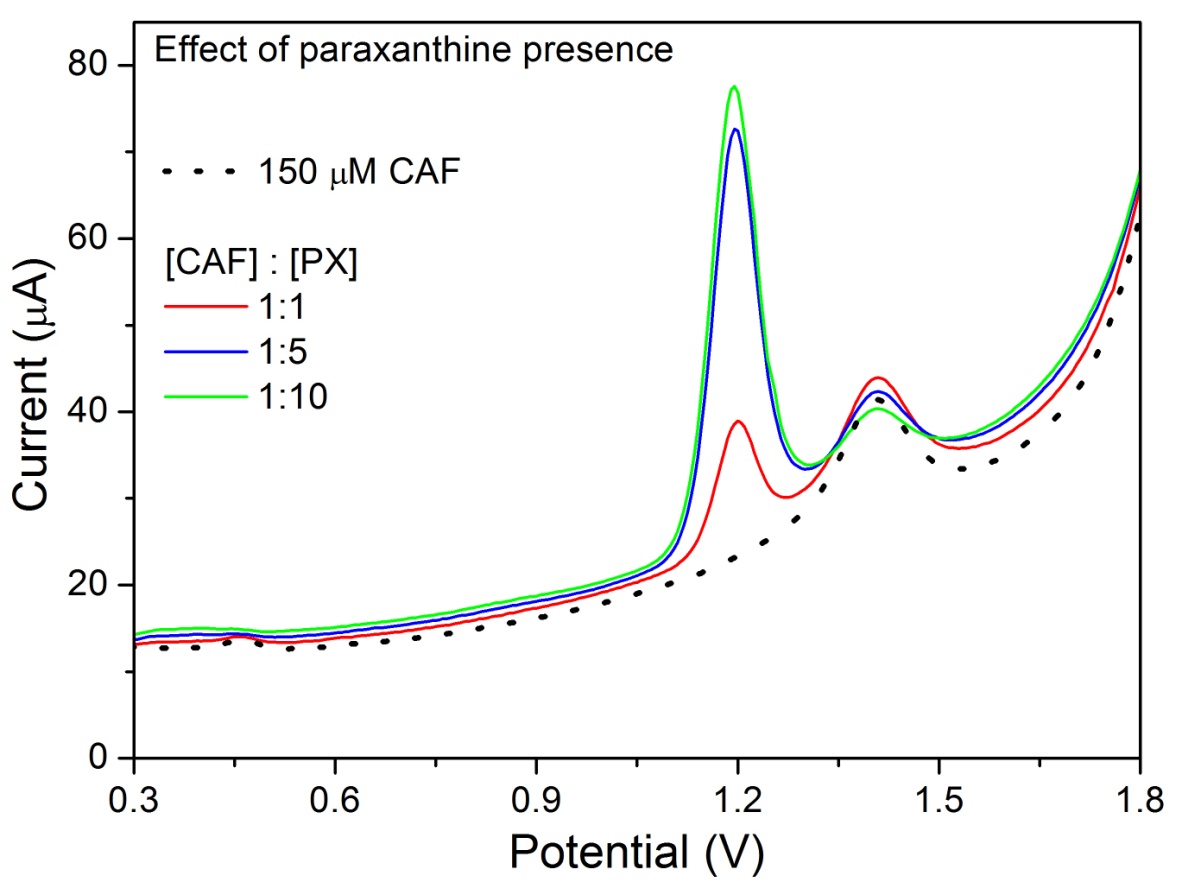
**

**Fig. S8.** DP voltammograms demonstrating the effect of the presence of paraxanthine (PX) on current response of 150 µM CAF in 0.1 M H_2_SO_4_ on GCE. The concentration ratios between CAF and PX are stated in the legend of figure. DPV parameters: pulse potential of 50 mV, pulse time of 10 ms and scan rate of 30 mV/s.

**
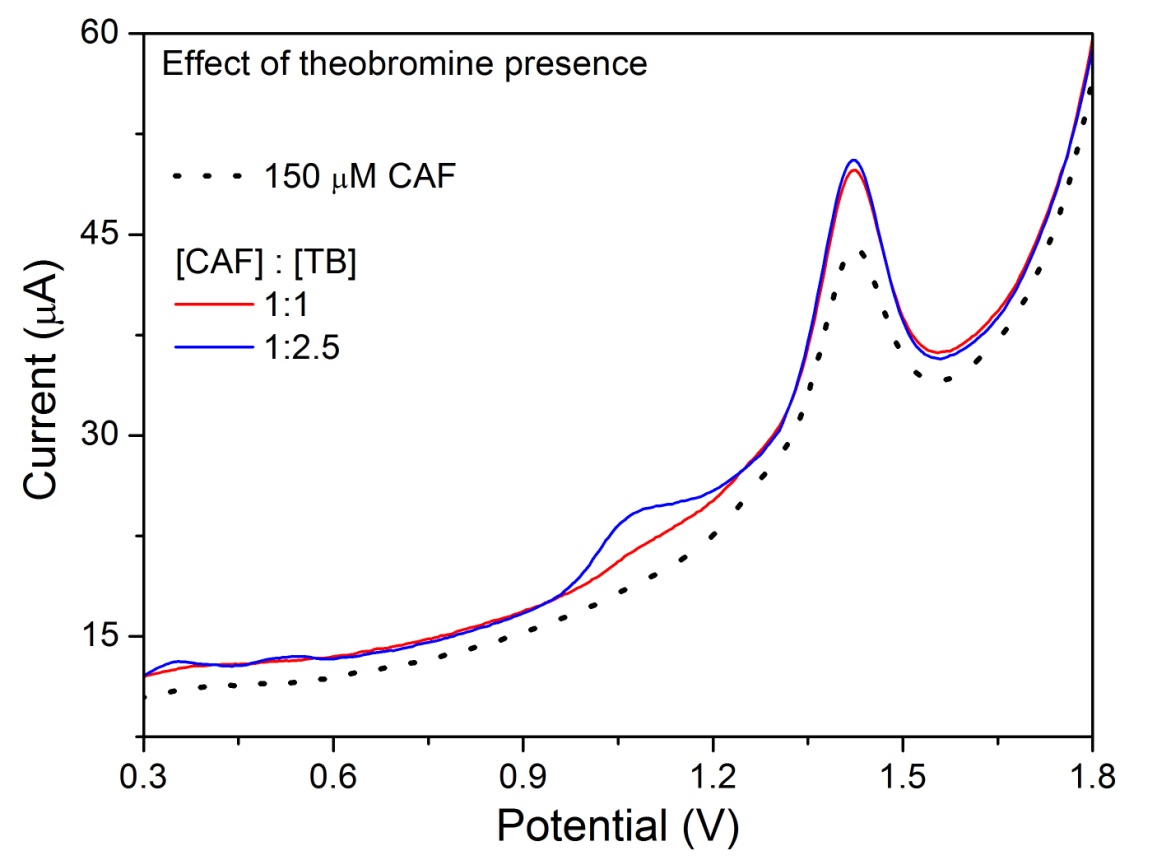
**

**Fig. S9.** DP voltammograms demonstrating the effect of the presence of theobromine (TB) on current response of 150 µM CAF in 0.1 M H_2_SO_4_ on GCE. The concentration ratios between CAF and TB are stated in the legend of figure. DPV parameters: pulse potential of 50 mV, pulse time of 10 ms and scan rate of 30 mV/s.


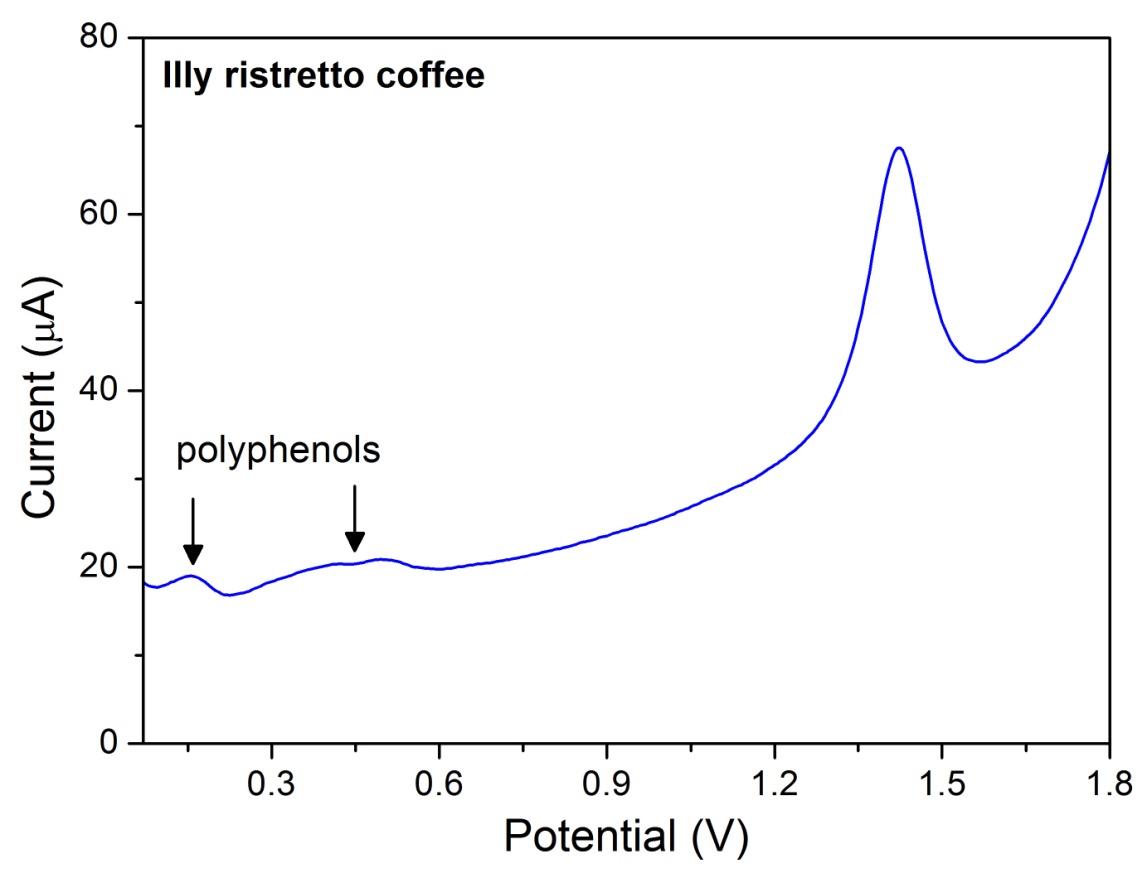


**Fig. S10.** DP voltammogram for coffee sample (*ristretto* espresso coffee) manifesting the presence of polyphenols in 0.1 M H_2_SO_4_ on GCE. DPV parameters: pulse potential of 50 mV, pulse time of 10 ms and scan rate of 30 mV/s.

**Fig. S11.** UHPLC chromatogram for analysis of Pepsi Cola with the clear and isolated signal for CAF observed at 5.305 min.


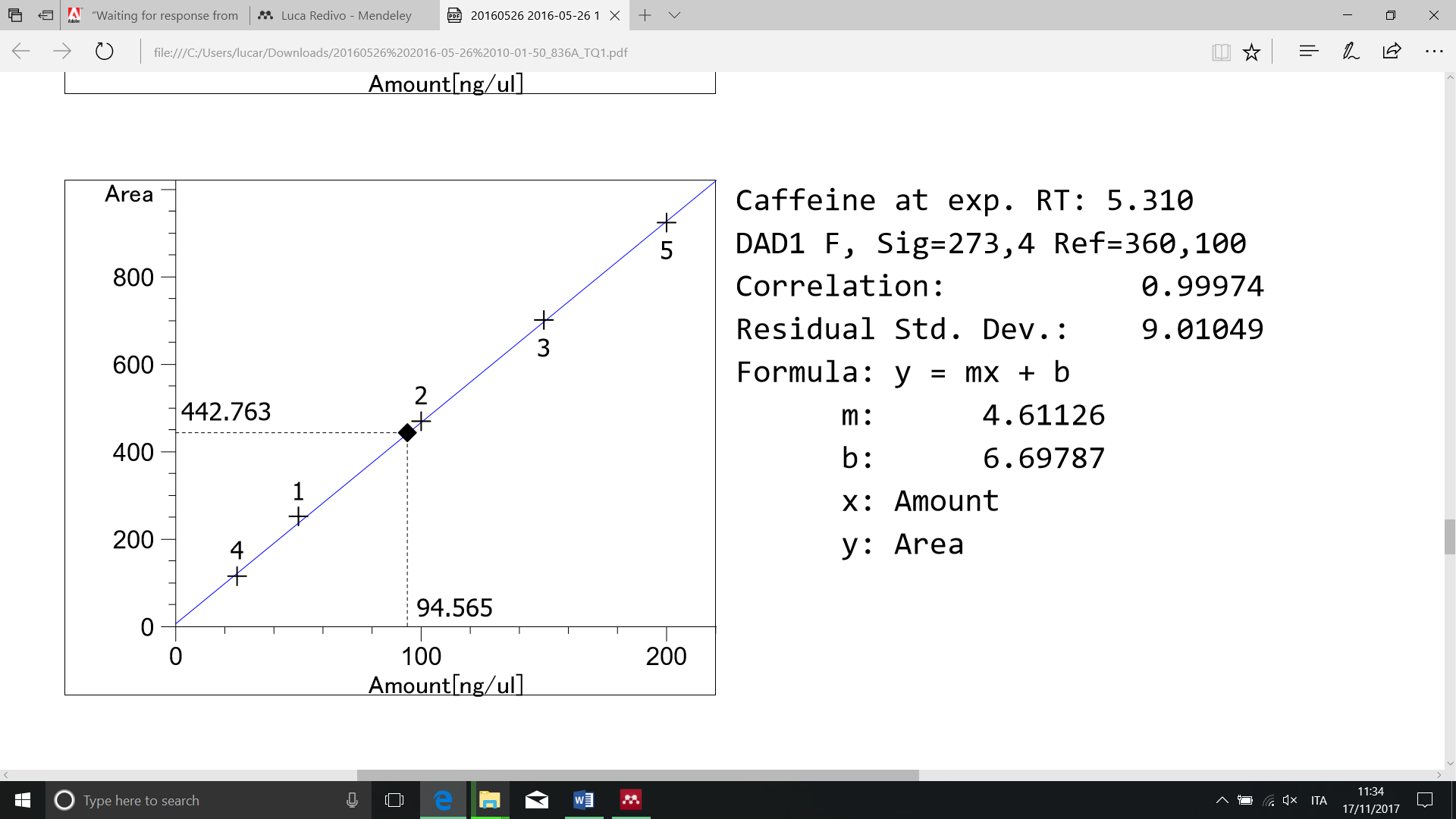


**Fig. S12.** Analysis of Pepsi Cola using chromatogram LC Open lab (Agilent Technologies, Waldbronn, Germany). The calibration curve employed for caffeine determination is reported in blue and the relative equation is reported on the side.
